# Inferring transcriptional modules from ChIP-chip, motif and microarray data

**DOI:** 10.1186/gb-2006-7-5-r37

**Published:** 2006-05-05

**Authors:** Karen Lemmens, Thomas Dhollander, Tijl De Bie, Pieter Monsieurs, Kristof Engelen, Bart Smets, Joris Winderickx, Bart De Moor, Kathleen Marchal

**Affiliations:** 1 BIOI@SCD, Department of Electrical Engineering, KU Leuven, Kasteelpark Arenberg, B-3001 Heverlee, Belgium; 2 Research Group on Quantitative Psychology, Department of Psychology, KU Leuven, Tiensestraat, B-3000 Leuven, Belgium; 3 Molecular Physiology of Plants and Micro-organisms Section, Biology Department, KU Leuven, Kasteelpark Arenberg, B-3001 Heverlee, Belgium; 4 CMPG, Department of Microbial and Molecular Systems, KU Leuven, Kasteelpark Arenberg, B-3001 Heverlee, Belgium

## Abstract

'ReMoDiscovery' is an intuitive algorithm to correlate regulatory programs with regulators and corresponding motifs to a set of co-expressed genes. It exploits in a concurrent way three independent data sources: ChIP-chip data, motif information and gene expression profiles. When compared to published module discovery algorithms, ReMoDiscovery is fast and easily tunable. We evaluated our method on yeast data, where it was shown to generate biologically meaningful findings and allowed the prediction of potential novel roles of transcriptional regulators.

## Abstract 2

ReMoDiscovery, a module discovery algorithm and software that uses ChIP-chip data, motif information and gene-expression profiles, is presented.

## Background

Complex cellular behavior is mediated by the action of regulatory networks. The reconstruction of these networks is one of the foremost challenges of current bioinformatics research [1,2] and requires combining different high throughput 'omics' data. With the current accuracy and availability of these high throughput data, the problem of network reconstruction remains highly underdetermined. The amount of independent experimental data is not sufficient to unequivocally estimate all parameters of the models. Previous studies, however, have unveiled that regulatory networks are modular and hierarchically organized [3]. Inferring modules instead of full networks drastically reduces the complexity of the inference problem and shows great promise for systems biology research [4]. A transcriptional network is reduced to a module consisting of a regulatory program and a corresponding set of co-expressed genes. The program, a set of regulators and their corresponding motifs, is responsible for the condition-dependent expression of the module's genes.

Traditionally, module identification methods dealt with each of the different 'omics' data sources separately (for example, solely based on microarrays [4]). However, simultaneous analysis of distinct data sources has a major advantage over their separate analysis: their integration allows gaining holistic insight into the network and a more refined definition of transcriptional modules can be derived [5]. Therefore, the more recent approaches for module inference combine several data sources.

Harbison *et al*. [6] and Kato *et al*. [7] both describe pragmatic approaches to analyze heterogeneous data. The approach by Segal *et al*. [4] focused on the identification of regulatory modules from microarray data with probabilistic models and was extended by Xu *et al*. [8] to incorporate ChIP-chip data. Tanay *et al*. [3] developed an advanced graph bicluster algorithm to simultaneously integrate expression data, ChIP-chip, protein interaction and phenotypic data. Bar-Joseph *et al*. [9] developed a procedure that learns modules from microarray and ChIP-chip data using a sequential analysis of the data. In a first step, the ChIP-chip data is used to find a set of genes whose upstream regions are likely to bind a common set of transcriptional regulators. In a second step, the microarray data is used to find a subset of this gene set, containing only those genes whose expression profiles are similar to each other. Finally, the resulting core set is expanded with additional genes that have a small combined *p *value for the same set of regulators in the ChIP-chip data.

In this paper, we present an alternative approach for module discovery based on heterogeneous data. It is different in spirit from previously suggested methods in that our algorithm takes distinct data sources related to transcriptional regulation, that is, microarray, ChIP-chip and motif data, into account in a concurrent (non-iterative or sequential) way. In contrast to previous methods, where motifs are mainly defined in a downstream analysis step, we use motif data as an independent information source. We demonstrate the performance of our method on well characterized yeast datasets.

## Results

**Figure 1:**
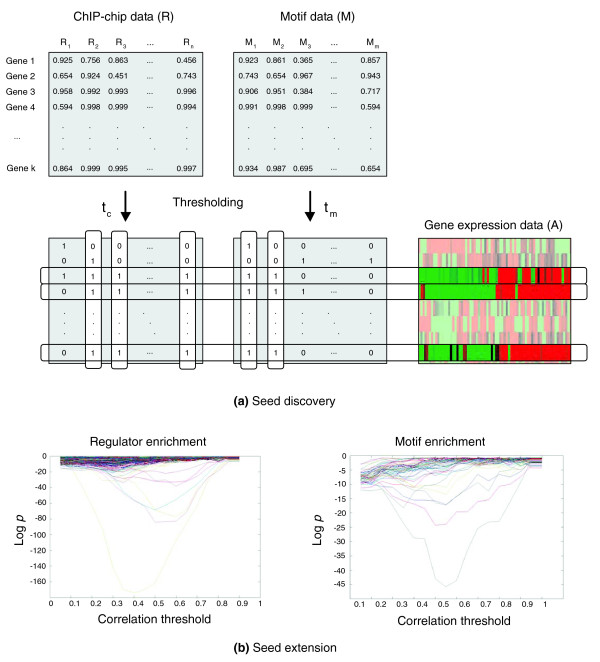
ReMoDiscovery analysis flow. ReMoDiscovery consists of a seed discovery step followed by a seed extension step. ChIP-chip data, motif data, and expression data are used as input for the algorithm. These three datasets can be represented as matrices in which the rows represent the genes. For the ChIP-chip data (R) the columns represent the regulators, for the motif data (M) they represent the motifs and for the expression data (A) the different experiments. **(a) **The seed discovery step identifies sets of genes that are co-expressed, bind the same regulators, and have the same motifs in their intergenic region. **(b) **The gene content of the seed modules can be extended during the seed extension step using less stringent criteria. The logarithms of the module enrichment *p *values (*y*-axis) are plotted for all regulators (motifs) as a function of the correlation threshold (*x*-axis). Each line in the sample plot shows the module enrichment *p *values for the enrichment of its corresponding regulator (motif) as a function of the gene expression correlation threshold used.

We aim at identifying transcriptional modules by searching microarray data for target genes with a common expression profile that also share the same regulatory program, based on evidence from ChIP-chip and motif data. Module detection by 'ReMoDiscovery' consists of two steps. In a first seed discovery step, stringent seed modules are identified (Figure 1). This seed discovery problem translates into finding gene sets (row dimension in Figure 1) that are co-expressed in microarray data (matrix M), that bind the same regulators (share the same columns in the ChIP-chip matrix) and that have the same motifs in their intergenic region (same columns in the motif matrix (Figure 1)). In a second seed extension step, the gene content of the module is extended using less stringent criteria. In the following, we discuss the specifics of this two-step procedure.

### Seed discovery step

In the seed discovery step, we detect large modules with tightly co-expressed genes (pairwise correlation of at least t_e_), directed by a common regulatory program with a minimum number of regulators (s_c_) and a minimum number of conserved motifs (s_m_) in the upstream region of the genes included in the module. Modules that meet these user-defined stringent criteria are defined as valid seed modules. We solely report 'maximal modules', defined as valid seed modules that become invalid upon extending them with any gene they do not yet contain.

An exhaustive search for all valid gene sets is not feasible, as the number of possible sets is exponential in the number of genes. However, by defining the constraints in such a way that extensions of an invalid module are never valid (that is, as hereditary constraints), we can adopt a fast Apriori-like algorithm to solve the problem [10] (see Materials and methods for details).

To determine the statistical significance of the obtained modules, we assigned a 'seed module' *p *value to each seed module (see Materials and methods). As expected, seeds with a high number of genes were highly significant. Modules with one gene were only significant if they contained many regulators.

To test the sensitivity of the seed discovery step with respect to the parameters, we compared results obtained at different parameter settings using a normalized Jaccard similarity score. The overall similarity in gene and regulator content was examined separately. We varied the correlation threshold on the expression profiles, the threshold on the ChIP-chip data t_c _(required to convert the ChIP-chip data to a binary matrix; see Materials and methods) and the minimum number of regulators s_c_. Parameter settings that are more similar generally resulted in more similar gene and regulator module content. This consistency (monotonicity) eases parameter tuning. Numerical results of the sensitivity analysis can be found on our supplementary ReMoDiscovery website [11].

### Seed extension step

The stringent criteria for the valid modules in the seed discovery step appear sufficient to reliably detect regulators and motifs, but the reported maximal gene content of such modules is likely to be underestimated in size. For this reason, ReMoDiscovery contains a second module extension step, in which the gene content of statistically significant seed modules is extended. This extension is performed by computing the module's mean expression profile, and ranking the remainder of the genes in the dataset according to their correlation with this seed profile. The genes at the top of the ranking will most likely belong to the module. However, it is not clear where to choose the cutoff on the correlation with the seed profile that is minimally required for additional genes to belong to the module. Therefore, 'module enrichment' *p *values are computed according to the enrichment of all regulators (motifs) in the extended modules as a function of the correlation cutoff. If motifs and regulators identified in the seed discovery step appear to be over-represented in the extended sets, the correlation resulting in the largest enrichment is considered optimal (Figure 1).

### Application to biological datasets

**Figure 2:**
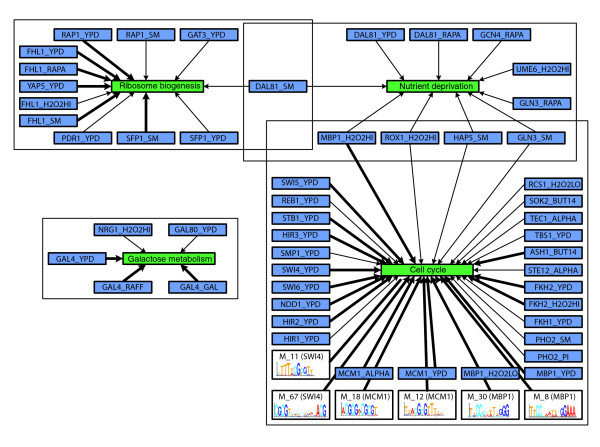
Overview of the seed modules identified in the Spellman dataset [12]. For visualization purposes, seed modules with similar function are combined (indicated in green). A regulator or motif that is part of a regulatory program of an extended module is indicated in the figure by a bold edge from the regulator or motif to its module.

We applied the algorithm described above to two well described yeast datasets: the Spellman dataset (assessing gene expression during cell cycle) [12] and the Gasch dataset (assessing gene expression in stress related conditions) [13]. Using the seed discovery step, we detected 20 seed modules for the Spellman dataset [12] and 104 seed modules for the Gasch dataset [13]. Detailed results can be found in Additional data files 1 and 2. Seed modules were all statistically significant when using a cutoff of 0.05 for the seed module *p *value. Significant seed modules that only contained one gene were omitted. To assess the biological relevance of the seed discovery step we compared our results with literature knowledge. We consider a seed module as verified if all of its regulators could be linked to the same biological process by the literature. For the Spellman dataset [12] 15 out of 20, and for the Gasch dataset [13] 53 out of 104 seed modules were supported by the literature. The seed modules for the Spellman dataset [12] are displayed in Figure 2, and those for the Gasch dataset [13] are presented in Additional data file 3. Part of the seed modules (18 out of 20 for the Spellman dataset [12]; 63 out of 104 for the Gasch dataset [13]) could be extended by the second step of the algorithm. The extended modules are described in detail in Additional data files 4 and 5 and all of their regulatory programs were found to be supported by the literature.

In some cases, seed modules could not be extended, that is, no additional correlated genes appeared to be present in the dataset under study. This implies either that the true module size was extremely small (only a few genes belong to the module) or that the module's regulatory program, although being biologically relevant, was not active in the conditions tested in the expression data. Indeed, the identification of the regulatory program in the seed discovery step is to a large extent determined by the ChIP-chip and motif data. However, motif data are condition independent. Sharing a motif thus does not necessarily imply co-expression in the tested microarray conditions. Similarly, because of the discrepancies in experimental conditions between available ChIP-chip and expression data, evidence from the ChIP-chip data does not automatically imply support by all microarray data. As a result, a module can only be extended with additional genes if its regulatory program appears to be active in the conditions underlying the used microarray study. Based on this observation, we subdivided modules into those involved in general metabolism found active in both datasets (for example, ribosome synthesis, galactose metabolism) and those related to processes for which the activity was restricted to either one of the datasets. To the latter group belong modules involved in the cell cycle, which were extended in the Spellman dataset [12], and modules related to nutrient deprivation, stress, respiration, amino acid metabolism, filamentous growth and meiosis extended in the Gasch dataset [13]. A more detailed description of the modules is given below.

### Detailed description of the detected modules

To summarize results, modules were combined if their respective regulatory programs were involved in the same biological process.

#### Modules involved in ribosome biogenesis

Modules involved in ribosome biogenesis are active in both the Spellman [12] and the Gasch [13] dataset. This could be expected as ribosome biogenesis is known to be tightly coupled to cell cycle progression as well as to environmental changes that affect growth rate. Different regulators were found to be associated with these ribosome related modules, including Arg80, Dal81, Fhl1, Gat3, Gts1, Mbp1, Mth1, Ndd1, Pdr1, Pho2/Bas2, Rap1, Rgm1, Rme1, Sfp1, Smp1, Swi4, and Yap5. Of these, Fhl1 and Rap1 were found in most modules. Consistently, both factors have been reported as main transcriptional regulators of ribosomal gene expression [14,15]. Also, Sfp1 and Rgm1 have been implicated in ribosome biogenesis and most recent data indicate that the former could act as a receiver of nutritional and stress derived signals [14,16,17].

To our knowledge, no data are available that may confirm a direct involvement of the other transcription factors in ribosomal gene expression. Nevertheless, the processes in which these factors are known to be involved can be linked to ribosome biogenesis. For instance, Arg80, Dal81 and Pho2/Bas2 all function in the sensing and metabolic control of essential nutrients such as amino acids and phosphate, and it is well established that ribosomal protein gene expression is directly related to availability of essential nutrients [14,18-21]. Another example is the transcriptional regulator Gat3, an uncharacterized member of the GATA family of transcription factors that controls the expression of nitrogen catabolic genes. The GATA factors are regulated by the Tor pathway, a pathway that also regulates the expression of genes involved in ribosome biogenesis [19].

#### Modules involved in galactose metabolism

Both the Spellman [12] and the Gasch [13] dataset revealed active modules controlling so-called GAL genes (for example, GAL3, GAL1, GAL7, GAL10), which encode proteins involved in galactose metabolism. These modules comprise the transcriptional regulators Gal4 and Gal80, which are key regulators of the galactose metabolism [22-24] and the transcriptional repressor Nrg1, which is known to mediate glucose repression of the GAL genes [25].

Some transcriptional regulators that were retained only from the Gasch [13] dataset point towards interactions between this module for galactose metabolism and modules for other processes, such as cell cycle control via Mbp1 (see also cell cycle module) [26] and amino acid metabolism via Met32 (see also amino acid module) [27]. In addition, the module for galactose metabolism contains the regulators Oaf1, Pip2 and Ume6, which are involved in the induction of peroxisomal genes participating in β-oxidation [28], potentially linking galactose metabolism to this process.

#### Cell cycle

Nine modules involved in cell cycle control were found to be active in the Spellman dataset [12]. The transcriptional regulators connected to these cell cycle related modules include components such as Swi4, Swi6, Mbp1 and Stb1, constituting the transcriptional complexes SBF and MBF, which operate during progression from G1 to S phase [29,30], as well as components involved in G2/M-specific transcription, such as Fkh1, Fkh2 and Ndd1 [31-33]. Further support for our analysis comes from the observation that other factors with a role in cell cycle regulation were also retrieved. These include the transcriptional repressor Xbp1, the corepressors Hir1, Hir2 and Hir3, and the transcription factors Pho2, Reb1 and Rcs1. Xbp1 is a repressor sharing homology with Swi4 and Mbp1 [34]. Pho2 is involved with the early G1 transcription factor Swi5 in the control of the HO gene [35]. Hir1, Hir2 and Hir3 are involved in cell cycle regulated transcription of histone genes [36,37]. The transcription factor Reb1 is known to bind with high affinity to a sequence upstream of *CLB2 *[38], a gene whose regulation is important for completion of the normal vegetative cell cycle. The regulator Rcs1 is involved in timing the budding event of the cell cycle [39]. Additional factors identified are Ash1, Sok2, Ste12 and Tec1. Their presence in our modules might link cell cycle to processes discussed below, like mating type switching [40] and the filamentous growth pathway (see also filamentous growth module) [41-43].

#### Nutrient deprivation

Six modules with transcriptional regulators that mediate control of target genes under nutrient deprived conditions were active in the Gasch dataset [13]. The regulators include Gat1, Dal81, Dal82, and Gln3, which are all involved in nitrogen catabolite repression [21,44,45], Gcn4, which is the main regulator in general amino acid control [46-48], Rtg3, which is a transcription factor involved in regulation of genes required for *de novo *biosynthesis of glutamine and glutamate [49], Fhl1, the forkhead factor that regulates ribosome biosynthesis in response to nutrient availability [15], and Hap2, a transcription factor of the tricarboxylic acid cycle [50]. The ChIP-chip data obtained after treatment with rapamycin were especially informative for identifying the different modules comprising this nutrient deprivation module. Rapamycin is known to inhibit Tor (target of rapamycin) protein kinases, which function in a nutrient-sensing signal transduction pathway. Consistently, the processes and regulators for this module all show connections to the Tor-mediated nutrient-sensing signal transduction pathway [49-52].

#### Stress related conditions

Twenty modules directing general and specific stress responses were identified and extended in the Gasch dataset [12]. These modules contain several transcriptional regulators and subsets of them are known to help fine-tune stress responses to particular conditions. The regulators Msn2 and Msn4 present in our modules are known key regulators of stress-responsive gene expression in yeast [53-55]. Several regulators identified by our analysis can be related to triggering responses upon oxidative stress, such as Skn7, Yap1, Hap5, Rox1, Hsf1, Nrg1, Pho2/Bas2 and Yap4/Cin5 [56-60]. A connection with oxidative stress may also exist for Sut1, a factor that, according to the literature, relieves hypoxic genes from repression by the Cyc8-Tup1 [61] co-repressor complex, which is recruited to many promoters via regulatory proteins such as Rox1 [62]. With regard to oxidative stress, links with other stress responses could also be derived. Indeed, Cup9 mediates copper resistance [63] while Yap6 confers resistance to cisplatin [64].

Some regulators present in the stress related module have been reported to be operative in aspects indirectly related to stress response, for instance, Ngr1, Rim101, Sok2 and Ume6 are linked by their roles in meiosis and sporulation (see also module for filamentation and meiosis) [65-68] and Xbp1 is a stress-induced transcriptional repressor of the cell cycle (see also cell cycle module) [69].

#### Respiration

The Gasch dataset [13] enabled the identification of an extendable module dedicated to respiration that includes the heme-responsive factor Hap1 and the subunits Hap2, Hap4, Hap5 of the heme-activated CCAAT-binding complex [70,71]. Two motifs, motif 7 (Esr2: GRRAAAWTTTTCACT) and 70 (CGCGnnnnnGGGS), of which the latter is defined as a 'new' motif by Kellis *et al*. [72], could be associated with this module.

#### Amino acid metabolism

The modules for amino acid metabolism were recovered upon analysis of the Gasch dataset [13]. Support for the validity of this module came from the presence of Dal81, a positive regulator of multiple nitrogen catabolite repression genes [21,44,45] and from the presence of Gcn4, the main regulator in the general amino acid control [46-48]. Also present was Leu3, a transcriptional regulator of genes involved in nitrogen assimilation and in biosynthetic pathways of branched-chain amino acids [73,74]. The regulators Cbf1, Met4 and Met 32 of our module have previously been shown to be required for the coordinated expression of the structural genes from the sulfur amino acid biosynthesis pathway [75,76].

Additional regulators present in this module may provide links to other regulatory programs. The presence of Rox1 and Skn7 can couple this network to the program for oxidative stress response (see also stress related module) [56,57], while Sfp1, Rap1, and Gcr2, a coactivator of Rap1 [77], reflect links with ribosome biosynthesis (see also module for ribosome biogenesis) [14,21,78].

#### Modules involved in filamentous growth

Five modules related to filamentous growth could be retrieved from the Gasch dataset [13]. The filamentous growth pathway induces a morphogenetic switch under adverse growth, such as nutrient deprivation. This switch induces the formation of pseudohyphae, which are believed to facilitate foraging for scarce nutrients [41-43]. Consistent with the literature, these modules included the regulators Ste12 and its interacting partners Dig1 and Tec1, as well as Sok2 and its downstream regulators Ash1 and Phd1 [41-43]. The regulator Nrg1, also present in our module, is known to function as a negative regulator of filamentous growth and as a repressor of FLO11, which encodes a cell surface glycoprotein required for filamentous growth [79] (see also galactose metabolism module).

Filamentous growth is known to be intimately linked to growth and cell cycle control. As such, it is not surprising that our analysis also retrieved for this module factors involved in cell cycle control, such as components of SBF and MBF, that is, Swi4 and Mbp1 [29,30] or Fkh2 [80] (see also cell cycle module). This close link to growth also explains the presence in our modules of Fhl1 [14,15], Hap1 [70,71] and Sut1 [61] as they all have important functions in determining the growth potential of yeast cells. Our analysis additionally retrieved Sko1, an important regulator allowing cells to cope with osmotic stress. Osmotic stress can, under some conditions, induce filamentous growth and as such the presence of Sko1 in our modules makes sense [81,82].

#### Modules involved in meiosis

Finally, we identified one extendable module in the Gasch dataset [13] that is regulated by Ume6 and Rap1. We refer to this module as being important for meiosis because the literature confirmed that Ume6 has a key regulator function in this process [83,84] while Rap1 is believed to control Ume1, a regulator that is required for the repression of early meiotic genes [84].

### Comparison with other module inference tools

To assess the differences between ReMoDiscovery and previously described algorithms for module detection, we applied some of the well known module detection tools to which we had access to a workable implementation (that is, SAMBA [3] and GRAM [9]) along with ReMoDiscovery on the combined Spellman (microarray) [12] and Harbison (ChIP-chip) [6] dataset.

We analyzed running times on these datasets at distinct parameter settings for each of the tested algorithms on an Intel Pentium 2 GHz laptop with 512 Mb RAM. Independent of the setting for the 'overlap prior factor', the SAMBA algorithm [3] was rather quick, with running times around three minutes. For parameter settings close to its defaults, ReMoDiscovery performed slightly better. Running times were on the order of one minute. In general, the speed of the Apriori algorithm is roughly proportional to the number of modules that satisfy the constraints. In contrast, running times of the GRAM algorithm [9] were prohibitive if the data contained genes with more than ten significant ChIP-chip interactions due to the exponential increase in the number of candidate core modules (see [9] for details). After filtering out those genes, running times decreased to about 20 minutes at the default parameter setting.

To compare the gene and regulator content between modules obtained by GRAM [9], SAMBA [3] and ReMoDiscovery, we used an unsupervised scoring scheme that considers gene content and regulator content separately (that is, the normalized Jaccard similarity score as defined in Materials and methods). Since parameter settings influence the module composition, we calculated normalized Jaccard similarity scores on the results for a number of parameter settings (see Materials and methods).

**Figure 3:**
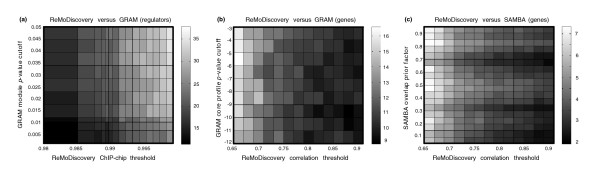
Representative examples from the module content similarity analysis. The significance of the similarity in module content between ReMoDiscovery seed modules and GRAM [9] and SAMBA [3] output is shown at different parameter settings. The color bar on the right indicates the normalized Jaccard similarity score, that is, the number of standard deviations from the mean of the distribution of Jaccard similarity scores on randomized module partitioning. **(a) **Regulator content similarity between ReMoDiscovery and GRAM, with varying GRAM module *p *value cutoff and ReMoDiscovery Chip-chip threshold. **(b) **Gene content similarity between ReMoDiscovery and GRAM, with varying GRAM core profile *p *value cutoff and ReMoDiscovery correlation threshold. **(c) **Gene content similarity between ReMoDiscovery and SAMBA, with varying SAMBA overlap prior factor and ReMoDiscovery correlation threshold.

For all settings, we observed that both the overlap in gene and regulator content between the GRAM [9] modules and the seed modules of ReMoDiscovery was highly significant (normalized Jaccard similarity scores around 15 and 25 standard deviations, respectively). Since GRAM [9] generally returns modules with less regulator content, the similarity in regulatory programs was best for the most stringent ReMoDiscovery ChIP-chip threshold (Figure 3). Accordingly, gene content was most similar if the ReMoDiscovery correlation threshold was lowered. From these results, we conclude that the ReMoDiscovery seed modules and the GRAM [9] modules represent similar patterns in the data, the former focusing on modules with fewer genes and more regulators, the latter on modules with more genes and less regulators. The regulatory programs discovered by SAMBA [3], using the discretization method suggested by the authors, did not significantly resemble those of ReMoDiscovery or GRAM [9]. The gene content on the other hand did show some overlap (Figure 3).

We performed a similar analysis, this time with the extended seed modules of ReMoDiscovery. Extending the seeds generally results in a smaller number of statistically overrepresented regulators in the modules (Table 1), but an increase in gene content. Accordingly, the scores for overlap in gene content with GRAM [9] and SAMBA [3] improved. The normalized Jaccard similarity score increased from 15 standard deviations to about 100 standard deviations for GRAM [9] and from 6 to about 21 standard deviations for SAMBA [3] (data not shown). At the same time, the regulator overlap with GRAM [9] increased to about 50 standard deviations. In other words, increasing the number of genes in a module (higher gene content) corresponded to decreasing the number of regulators (lower regulator content), making the results even more similar to those of GRAM [9].

All tools discussed in this study serve two purposes: they simultaneously identify clusters of co-expressed genes and the corresponding regulatory programs. To evaluate the first aspect, we calculated the average functional overrepresentation of the modules detected by each of the tools on our benchmark dataset (Table 1). We used default parameter settings for GRAM [9], SAMBA [3] and ReMoDiscovery. From these results it appears that, for all tools, regulatory modules are well enriched for known functional classes. For ReMoDiscovery, the enrichment score improved significantly upon extension of the seeds.

To test the sensitivity of these tools in retrieving regulators known to be involved in the cell cycle, we compiled a list of known regulators (see Materials and methods) and tested how many of these occurred in the regulatory programs of any of the cell cycle related modules (Table 2). We also displayed the ratios of the number of known cell cycle regulators over the total number of regulators detected in a module's program, averaged over all modules. These results show that both ReMoDiscovery and GRAM [9] had a considerably higher sensitivity than SAMBA [3] in retrieving cell cycle related regulators.

Conclusions about specificity should be treated with care because, in the absence of a golden standard (that is, a completely characterized network of interactions), the number of false positive predictions can never be quantified. Although the regulatory programs of GRAM [9] and ReMoDiscovery seem to be more enriched in cell cycle related regulators (larger ratio of known cell cycle related regulators over the total number of regulators than SAMBA [3]), it is not possible to distinguish between true and false positives without further experimental validation.

## Discussion

In this study, we present a two-step methodology to unravel active modules based on the concurrent analysis of three independently acquired data sources. The seed discovery step predicts putative seed modules (consisting of genes, regulators and corresponding motifs). The seed extension step optimizes the gene content of the modules and indicates whether the seed modules' regulatory program is active in the microarray data.

The data integration problem is tackled in a very direct way: using the Apriori algorithm, no iteration over the different data sources is required. As regards the algorithmic properties, a comparison of ReMoDiscovery with other module detection tools revealed that speed is one of the major advantages of the Apriori strategy. ReMoDiscovery's running times and memory requirements are drastically smaller than those of certain other module detection algorithms such as the GRAM [9] algorithm. This is important as most module discovery algorithms require repeated testing to find the optimal parameter settings. Together with the straightforward biological interpretation of the parameters, its speed turns ReMoDiscovery into a user-friendly, readily tunable tool.

The biological relevance of our method was assessed by applying it on the extensively studied Spellman [12] and Gasch [13] datasets. Comparison of our results with the literature showed that experimental evidence existed for many of our statistically significant seed modules. For modules for which no direct evidence existed so far, a plausible explanation for their composition could very often be inferred from the literature and potential new links between the detected pathways and modules could be derived. A seed module that can be extended with more genes in the seed extension step gives a clue to the regulatory program being active in the prevailing conditions of the tested microarray experiment. Based on this observation, a distinction could be made between modules involved in general metabolism that were active in both datasets (for instance, ribosome synthesis, galactose metabolism) and the more specialized modules (for instance, cell cycle, nutrient deprived conditions, stress related conditions, amino acid metabolism, respiration, filamentous growth or meiosis) for which the activity was restricted to either one of the datasets.

In contrast to previous approaches in which motif information results from downstream analysis of the inferred modules, our method used this information as an independent input source. To avoid circular reasoning, we ensured that motif information was derived from sequence information only and did not rely on any other experimental data source (for instance, as available in the motif compendium of Kellis *et al*. [72]). Therefore, the compendium of motifs we used as an input dataset is far from complete. This explains why we detected less motifs for each module compared to, for instance, Kato *et al*. [7] or Harbison *et al*. [6].

To assess to what extent ReMoDiscovery discovers modules similar to those detected by other module identification tools, we compared it with previously described tools on the same benchmark set. Compared to GRAM [9], we found a significant overlap in both gene and regulator content of the detected modules over a sweep of different parameters. The similarity between both algorithms was larger when comparing the results of GRAM [9] with those of the extended seed modules than with the original seed modules. This difference reflects the trade-off between the number of regulators and the number of genes in biological modules: modules comprising a regulatory program with many regulators (such as our seed modules) can be expected to contain few genes with a potentially highly related function. In a module, the number of genes will usually increase with a decreasing number of regulators. Obviously, there will be more genes that only share part of their regulatory program, that is, the part that is active under the tested set of conditions. While our seed modules give a view on the complete regulatory program, our extended modules highlight the program active in the microarray dataset. They contain more genes and are more similar to the GRAM [9] output. Hence, with ReMoDiscovery we offer an algorithm that can be used to focus on very specific regulatory programs (seed modules) as well as on less specific modules with more genes (extended seed modules). The most appropriate choice depends on the specific research question under study, so usually there is no single best solution for the outcome of a module detection algorithm.

In our hands, the regulatory programs found with the SAMBA algorithm [3] did not significantly resemble those of ReMoDiscovery or GRAM [9]. The possibility might exist that SAMBA [3] focuses on other aspects of the data and, therefore, detects fundamentally different modules. However, based on our analysis we believe that the regulatory programs recovered by SAMBA [3] are unlikely to be biologically meaningful as the sensitivity in detecting cell cycle related regulators was low. Most likely the available download of the SAMBA-Expander application [3] is not yet fully adjusted to the use of heterogeneous data sources.

## Conclusion

We developed an intuitive algorithm for the automatic inference of transcriptional modules. It is fast, readily tunable and flexible, in the sense that it can easily be extended to include other information sources, as long as the constraints on the gene sets are hereditary. Our method does not require large microarray compendia but allows for an easy first screen of transcriptional modules being active or present in one's own 'small' microarray dataset, using publicly available ChIP-chip and motif data. In principle, our method is generic and applicable for all organisms for which the three data sources are available. However, its sensitivity will be largely determined by the completeness of ChIP-chip and motif data, which are expected to improve over time.

## Materials and methods

### Microarray data

The Spellman [12] and Gasch [12] datasets were used as microarray benchmark sets. The Spellman dataset [12] contains 77 experiments describing the dynamic changes of yeast genes during the cell cycle. The Gasch dataset [13] consists of 177 experiments, examining gene expression behavior during various stress conditions. Expression profiles were normalized (subtracting the mean of each profile and dividing by the standard deviation across the time points) and stored in a gene expression data matrix, denoted by A, with a row for each gene expression profile and a column for each condition.

### Location data

Genome-wide location data performed by Harbison *et al*. [6] were downloaded from their website [85]. These contain information regarding the binding of 204 regulators (although Harbison *et al*. [6] only describe 203 regulators) to their respective target genes in rich medium (the 106 regulators initially profiled by Lee *et al*. [86] and 98 new regulators). Besides rich medium, 84 regulators were profiled in at least one environmental condition other than rich medium.

For ReMoDiscovery, the ChIP-chip data matrix (denoted by R) consists of one minus the 'ChIP-chip *p *values' for each gene, obtained from the combined ratios of immuno-precipitated and control DNA using an error model (see Harbison *et al*. [6]). Both GRAM [9] and SAMBA [3] use ChIP-chip *p *values and require some additional preprocessing. As the authors of GRAM [9] suggested, genes that bind more than 10 regulators (ChIP-chip *p *value < 0.001) were omitted. For SAMBA [3], we transformed all ChIP-chip data to a log10 scale, nullified all values above 0.02 and used a parametric discretization setting in the Expander software tool according to the authors' advice.

### Motif data

The motif data used in this study were obtained from a comparative genome analysis between distinct yeast species (phylogenetic shadowing) performed by Kellis *et al*. [72]. These motifs, available online as regular expressions, were transformed into their corresponding weight matrices (see online information for more details [11]). Out of the 71 putative motifs described by Kellis *et al*. [72], the 53 most informative ones were retained. The weight matrices corresponding to these motifs was subsequently used to screen all intergenic sequences of yeast using MotifLocator [87]. The higher the score of a motif hit in a gene, the more likely it will be a true instance. Results of the screening can thus be summarized in a matrix M that contains for each gene-motif combination a score that indicates how likely it is the gene contains an instance of the respective motif.

### Algorithm for seed discovery

The algorithmic details of our method are based on the observation that the particular choice of the constraints guarantees that, given an invalid module, none of its extensions can ever become valid. For this reason, we call the constraint set 'hereditary'. Such a hereditary constraint set has first been deployed in the so-called Apriori algorithm, which is described in a seminal paper by Agrawal and Imielensky [10]. In ReMoDiscovery, the constraints are the minimum number of regulators (or regulator support constraint s_c_), the minimum number of motifs (or motif support constraint s_m_), and a minimal pairwise correlation between genes in a module t_e_. We apply these constraints to find regulatory modules that contain as many genes as possible. Since the regulator binding and motif data consist of non-binary score values, the support values are estimated by using thresholded regulator and motif scores, equal to 1 if the score is larger than a threshold t_c _or t_m_, respectively, and 0 otherwise. After thresholding, the regulator and motif data are binary, and are represented in the matrices R and M (Figure 1). Note also that the current implementation uses correlation as a measure for co-expression. If required, however, other similarity measures could be used in the Apriori framework.

Using the hereditary constraints results in a significant speed-up with respect to a naïve exploration of the space of possible modules, because we do not need to explicitly check large gene sets for validity. Each subset of genes of a valid module necessarily represents a valid module. This fact can be exploited to reduce the number of times the constraints need to be evaluated. Indeed, only gene sets for which all subsets have been found to be valid modules need to be checked, and they can be discarded *a priori *if one of their subsets turns out to be invalid, even before checking the constraints. In summary, a high level description of the algorithm is: first, choose parameter values s_c_, s_m_, t_e_, t_m _and t_c_; second, threshold the regulator and motif data using thresholds t_m _and t_c_, yielding the binary tables R and M; third, find all maximal modules for which the support constraints specified by s_c _and s_m _are satisfied, and for which the correlation between the gene expression profiles of any pair of genes in the module exceeds the required threshold t_e_; and fourth, report maximal modules along with the motifs and regulators that support them.

To assess statistical significance, we assigned a seed module *p *value to each module obtained at a specific parameter setting. To this end, we randomly permuted the gene labels for each dataset (ChIP-chip, motif data, gene expression) separately. This randomization procedure was repeated 100 times. The results of ReMoDiscovery seed discovery on these random datasets were used to construct an empirical joint distribution on the number of regulators and genes from which we calculated a seed module *p *value for each of the seeds found in the real data sets.

### Module extension: calculate enrichment of motifs and regulators

To determine the module enrichment *p *value for the enrichment of a particular motif (regulator) in an extended module with *n *genes, we first calculated the mean score of that motif in the module by averaging out the entries in the original motif (regulator) data matrix in the column corresponding to the motif (regulator) and the rows corresponding to genes in the module. We then compared this mean score to the distribution of scores obtained on a random selection of *n *genes, for the same motif (regulator). Note that the mean score of a module by random gene selection is approximately Gaussianly distributed (central limit theorem), with mean equal to the mean over all genes, and variance equal to the overall variance divided by the size of the module. This Gaussian approximation of the H_0_-hypothesis is used to calculate a module enrichment *p *value for a particular motif or regulator.

### Application of ReMoDiscovery to the yeast data

The total data matrix used consisted of 6,144 genes (that is, the intersection of the number of rows of the motif, ChIP-chip and microarray matrices). When applying our algorithm to the yeast dataset, we used the default parameters, that is, the motif threshold t_m _equaled 0.9, the ChIP-chip threshold t_c _was 0.99, and the correlation threshold t_e _was 0.75. The minimal number of motifs s_m _was set to 1 such that we find seed modules that have at least 1 motif in their regulatory program. We varied the minimal number of regulators s_c _over the values 3, 4, 6, 8 and 10 for the Spellman [12] dataset and over 4, 6, 8 and 10 for the Gasch [13] dataset. Resulting seed modules with a seed module *p *value > 0.05 were evaluated during the second seed extension step.

### Comparison with other methods

We downloaded the java implementation of the SAMBA [3] software package from [88]. The Matlab code of the GRAM algorithm [9] was obtained from the authors upon request.

We used ReMoDiscovery with a ChIP-chip threshold (one minus the ChIP-chip *p *value) equal to 0.99, a correlation threshold of 0.75 and a minimum of one motif and four regulators, respectively. When comparing regulator content from ReMoDiscovery to SAMBA [3] and GRAM [9], we looked at the ReMoDiscovery seed modules for a minimum number of regulators equal to 3, 4, 6, 8 and 10. We also examined the influence of a variation in ChIP-chip threshold, in the range [0.98 to 0.999] (values below 0.98 were not tested as the quality of the biological outcome started to decrease). The SAMBA [3] 'overlap prior factor' was varied between 0 and 1, in steps of 0.05. The latter parameter describes the extent of overlap that is permitted between different modules in the same solution. For the GRAM algorithm [9], we varied all user defined parameters in a wide range: the base 10 logarithm of the 'core profile *p *value cutoff' between minus 12 and minus 3, the 'num in core cutoff' between 5 and 97 and 'module *p *value cutoff' between 0.001 and 0.05. When comparing gene content from ReMoDiscovery to SAMBA [3] and GRAM [9], we considered ReMoDiscovery output for varying correlation threshold, in the range (0.65 to 0.9).

Module comparison was based on the normalized Jaccard similarity score [89]. For a specific parameter setting, we verify for each pair of genes (regulators) whether these two genes (regulators) occur together in at least one module. Doing so for all gene (regulator) pairs and for both methods, one can define the number of true positives TP as the number of gene pairs occurring together at least once in both methods. Analogously, the number of false positives FP, true negatives TN and false negatives FN can be defined. As in [89], we used the Jaccard similarity score TP/(TP + FP + FN) to score the overlap between two module compositions. In addition, randomizations were used to determine the significance of a specific score. This leads to the notion of normalized similarity scores, expressed as the number of standard deviations from the mean of the distribution of Jaccard similarity scores for randomized module compositions. For a more detailed description of our module comparison approach, we refer to our supplementary website [11].

### Evaluating the statistical significance for functional category enrichment of modules

The hypergeometric distribution was used to determine which functional categories were statistically overrepresented in the extended modules. For each module we computed the fraction of genes associated with each functional category in the MIPS database [90] and used the hypergeometric distribution to calculate a corresponding 'functional enrichment *p *value'. Modules with a functional enrichment *p *value below 0.05 (no compensation for multiple testing) were considered significantly enriched.

### List of cell cycle regulators

We compiled a list containing every regulator that was present in the regulatory program of at least one cell cycle enriched module identified by ReMoDiscovery, GRAM [9] or SAMBA [3]. The regulators in this list that are involved in cell cycle according to the Saccharomyces Genome Database [91] were considered 'cell cycle regulators': ACE2_YPD, FKH1_YPD, FKH2_H2O2Hi, FKH2_H2O2Lo, FKH2_YPD, MBF1_YPD, MBP1_H2O2Hi, MBP1_H2O2Lo, MBP1_YPD, MCM1_Alpha, MCM1_YPD, NDD1_YPD, RFX1_YPD, RPN4_YPD, STB1_YPD, SWI4_YPD, SWI5_YPD SWI6_YPD, YOX1_YPD (nomenclature adopted from Harbison *et al*. [6]). We used this list of 19 regulators to calculate the method's sensitivities.

### Other software

Networks were drawn using Cytoscape [92].

## Additional data files

The following additional data are available with the online version of the paper. Additional data file 1 and Additional data file 2 contain the seed modules for the Spellman [12] and Gasch [13] datasets, respectively. Additional data file 3 gives a graphical overview of the seed modules identified in the Gasch [13] dataset. Additional data file 4 and Additional data file 5 consist of the extended modules identified in the Spellman [12] and Gasch [13] datasets, respectively. Additional data file 6 includes the stand-alone version of ReMoDiscovery and a corresponding ReMoDiscovery help file.

## Supplementary Material

Additional File 1 The seed modules for the Spellman [12] dataset Click here for file

Additional File 2 The seed modules for the Gasch [13] dataset Click here for file

Additional File 3 Graphical overview of the seed modules identified in the Gasch [13] dataset Click here for file

Additional File 4 The extended modules identified in the Spellman [12] dataset Click here for file

Additional File 5 The extended modules identified in the Gasch [13] dataset Click here for file

Additional File 6 Stand-alone version of ReMoDiscovery and a corresponding ReMoDiscovery help file Click here for file

## Figures and Tables

**Table 1:** Summary of the results of the GRAM, SAMBA and ReMoDiscovery module discovery methods

Method	No.	Genes				Regulatory program		
			
		Mean	Min	Max	Mean functional enrichment	Mean	Min	Max
ReMoDiscovery (seed modules)	20	2.05	2	3	0.05	6.15	3	12
ReMoDiscovery (extended modules)	18	67.72	6	200	2.00E-03	3.50	2	6
GRAM	274	6.80	5	33	0,02	2.35	1	8
SAMBA	205	57.53	5	265	1.10E-02	4.16	0	31

The number of modules (No.) and the mean (Mean), minimum (Min) and maximum (Max) number of genes and regulators in the identified modules are displayed, as well as the average functional enrichment of the modules (Mean functional enrichment).

**Table 2:** Summary of the significantly cell cycle enriched modules, identified by the GRAM, SAMBA and ReMoDiscovery module discovery methods

Method	No.	Genes			Regulatory program					
			
		Mean	Min	Max	Mean	Min	Max	No. cell cycle R/all R	No. non cell cycle R/all R	No. cell cycle R
ReMoDiscovery (seed modules)	2	2	2	2	4	3	5	0.80	0.20	6
ReMoDiscovery (extended modules)	8	97.38	12	200	3.50	2	6	0.92	0.08	10
GRAM	33	6;47	5	11	2.66	1	6	0.74	0.26	17
SAMBA	14	58,;29	17	155	2.57	0	12	0.29	0.71	5

The number of cell cycle modules (No.) and the mean (Mean), minimum (Min) and maximum (Max) number of genes and regulators in these modules are displayed. Additionally, the ratio of the number of cell cycle regulators over the total number of regulators in a module, averaged over all cell cycle modules (No. cell cycle R/all R) is shown, as well as the ratio of the number of non-cell cycle regulators over the total number of regulators in a module, averaged over all cell cycle modules (No. non-cell cycle R/all R). The last column contains the number of regulators from the compiled list of 19 known cell cycle regulators (see Materials and methods) that were present in the regulatory program of at least one of the cell cycle modules (No. cell cycle R).
