# Supplementary material for: Inferring transcriptional modules from ChIP-chip, motif and microarray data
Source: Genome Biol. 2006 May 5;7(5):R37. doi: 10.1186/gb-2006-7-5-r37 (PMC1779513; doi:10.1186/gb-2006-7-5-r37)
Supplement: Additional File 6 — Stand-alone version of ReMoDiscovery and a corresponding ReMoDiscovery help file [file gb-2006-7-5-r37-S6.zip › ReMoDiscovery/readme.pdf]

REMODISCOVERY

ReMoDiscovery is free to use for academic purposes only. If used in your academic work, please include all relevant references by its authors. For any non-academic purpose, please contact its authors.
In addition, ReMoDiscovery can be distributed freely for academic purposes, as long as this file and all other files mentioned in this file are distributed along with the code.

ReMoDiscovery makes use of JFreeChart and JCommon (http://www.jfree.org/), the original jar files of which are also included in this distribution. They should always be distributed along with ReMoDiscovery, as well as this reference to JFreeChart and JCommon. By using ReMoDiscovery you agree to adhere to the terms of the GNU Lesser General Public License (LGPL) to which JFreeChart and JCommon are subject. The GNU Lesser General Public License (LGPL) is included in this distribution, and should remain so, if further distributed: LGPL-license.txt.

Please direct comments and questions related to the software to: tijl.debie@gmail.com or Kathleen.marchal@biw.kuleuven.be

Also check the accompanying website for recent updates (http://homes.esat.kuleuven.be/~kmarchal/Supplementary_Information_Lemmens_2006/Index.html)

INSTRUCTIONS

Unpack the zip-file ReMoDiscovery.zip and store all files in the same folder.
Make sure java 1.5 is installed.

Then:
- In windows: double-click on ‘ReMoDiscovery.bat’.
- Otherwise: execute the command ‘java -jar -Xmx512m remodiscovery.jar’ in a terminal, in the folder in which the files are stored.

The following window appears:

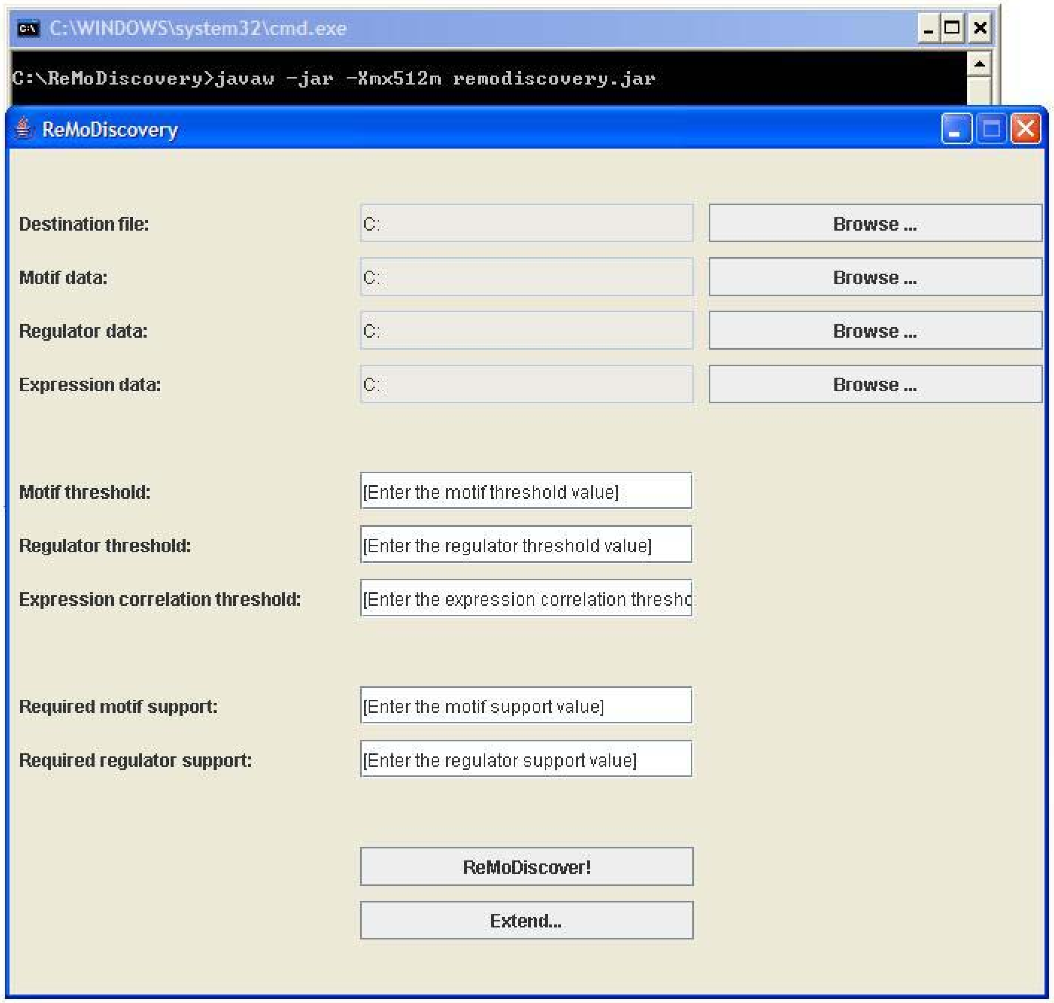

Output file (e.g. output.txt):

- Destination file: this file will contain the genes, regulators and motifs that are present in the seed modules.

Input files (Motif data (e.g. M.tx), Regulator data (e.g. C.tx), Expression data (e.g. A.txt)):

The input files should be of the following format: the rows represent the genes, and for each of the respective input files the columns represent the motifs (motif data), the regulators (ChIP-chip data) or the experiments (expression data). Each input file should contain the same number of genes, ordered in the same way. Both the rows and the columns should be numbered in the input file. For example files see the accompanying website .

Parameters:

- Motif threshold: the minimum score that a motif should have.
- Regulator threshold: the minimum score that a regulator should have.
- Expression correlation threshold: the minimum correlation between the expression profiles of two genes.

- Required motif support: the minimum number of motifs that should be shared by the genes in a module.
- Required regulator support: the minimum number of regulators that should be shared by the genes in a module.

Upload the correct input files and set the parameters. Subsequently click “ReMoDiscover!”. This will perform the first step of the algorithm, i.e. the seed discovery step.

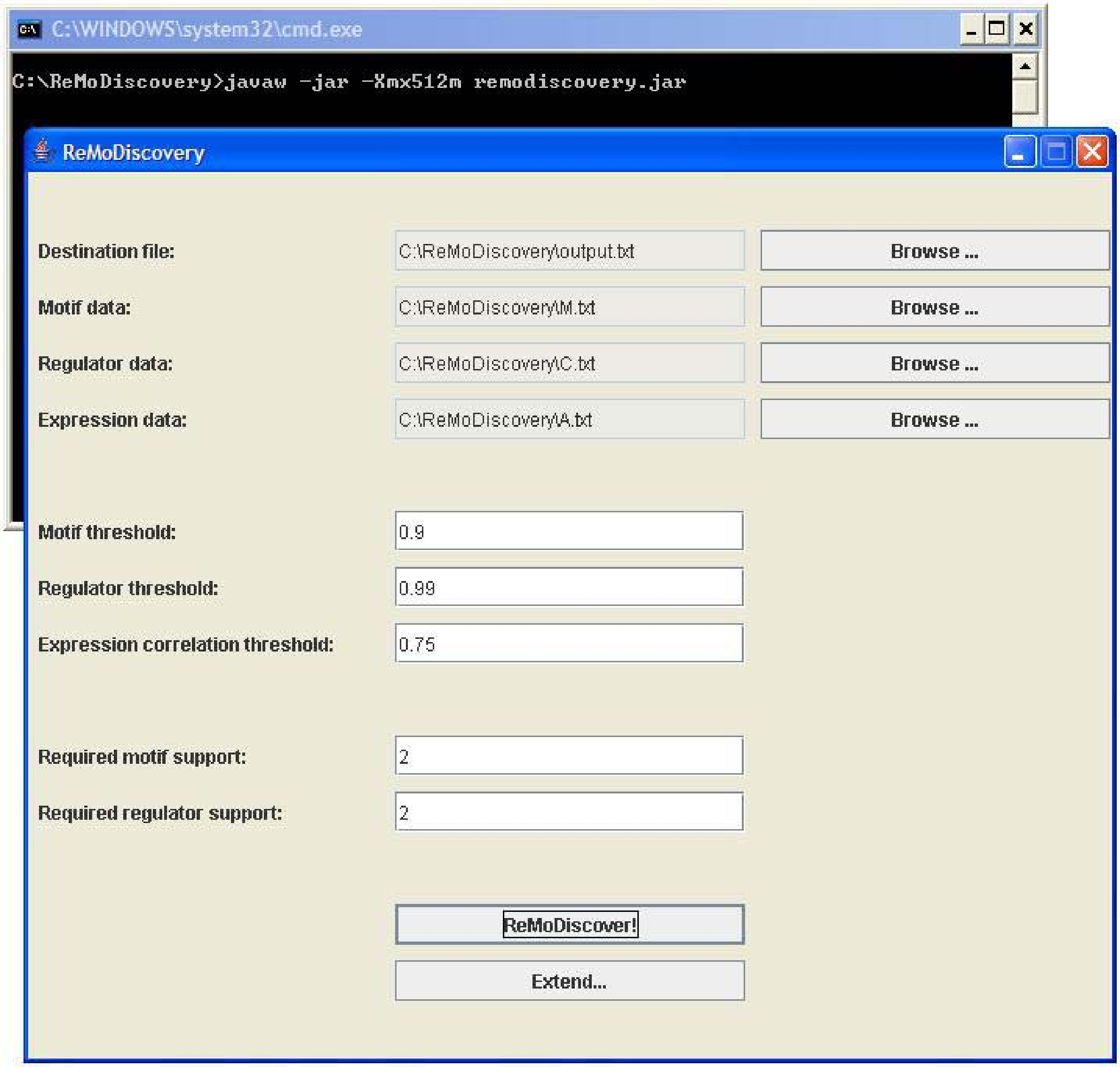

The algorithm will find sets of genes (seed modules) that satisfy the constraints. The identified seed modules can be found in the “Destination file”. An example of a “Destination file” is given below: each row in the file represents an identified seed module which is characterised by the indices of the genes belonging to the module e.g. the first module is a singleton containing gene 10, motifs M_5 and M_66 and regulators R_11 and R_46.

```
Genes:     10
Motifs:    M_5       M_66
Regulators:          R_11      R_46

Genes:     11
Motifs:    M_29      M_30      M_36      M_38      M_43      M_61      M_71
Regulators:          R_57      R_62      R_86

Genes:     13
Motifs:    M_1       M_29
Regulators:          R_1       R_87

Genes:     23
Motifs:    M_1       M_5       M_15      M_29
Regulators:          R_1       R_11      R_22

Genes:     28
Motifs:    M_1       M_10      M_14      M_21      M_22      M_31      M_51      M_57
Regulators:          R_1       R_97

…

Genes:     4451      6018
Motifs:    M_2       M_18
Regulators:          R_53      R_100

Genes:     4860      5855
Motifs:    M_10      M_47
Regulators:          R_13      R_107

Genes:     5545      5855
Motifs:    M_7       M_37
Regulators:          R_78      R_79      R_107

Genes:     119       2135      4347
Motifs:    M_11      M_12      M_21
Regulators:          R_93      R_98

Genes:     556       4451      4991
Motifs:    M_11      M_18
Regulators:          R_53      R_100

Genes:     610       1151      4796
Motifs:    M_16      M_30      M_59
Regulators:          R_23      R_54      R_64

Genes:     1248      1757      3664
Motifs:    M_14      M_16
Regulators:          R_2       R_23

Genes:     1918      5148      5971
Motifs:    M_8       M_11
Regulators:          R_23      R_98


Genes:     556       991       4991      5530
Motifs:    M_12      M_18
Regulators:          R_53      R_100
```

Once the first step of the algorithm is completed, the second step, i.e. the seed extension step, can be performed by clicking "Extend…".

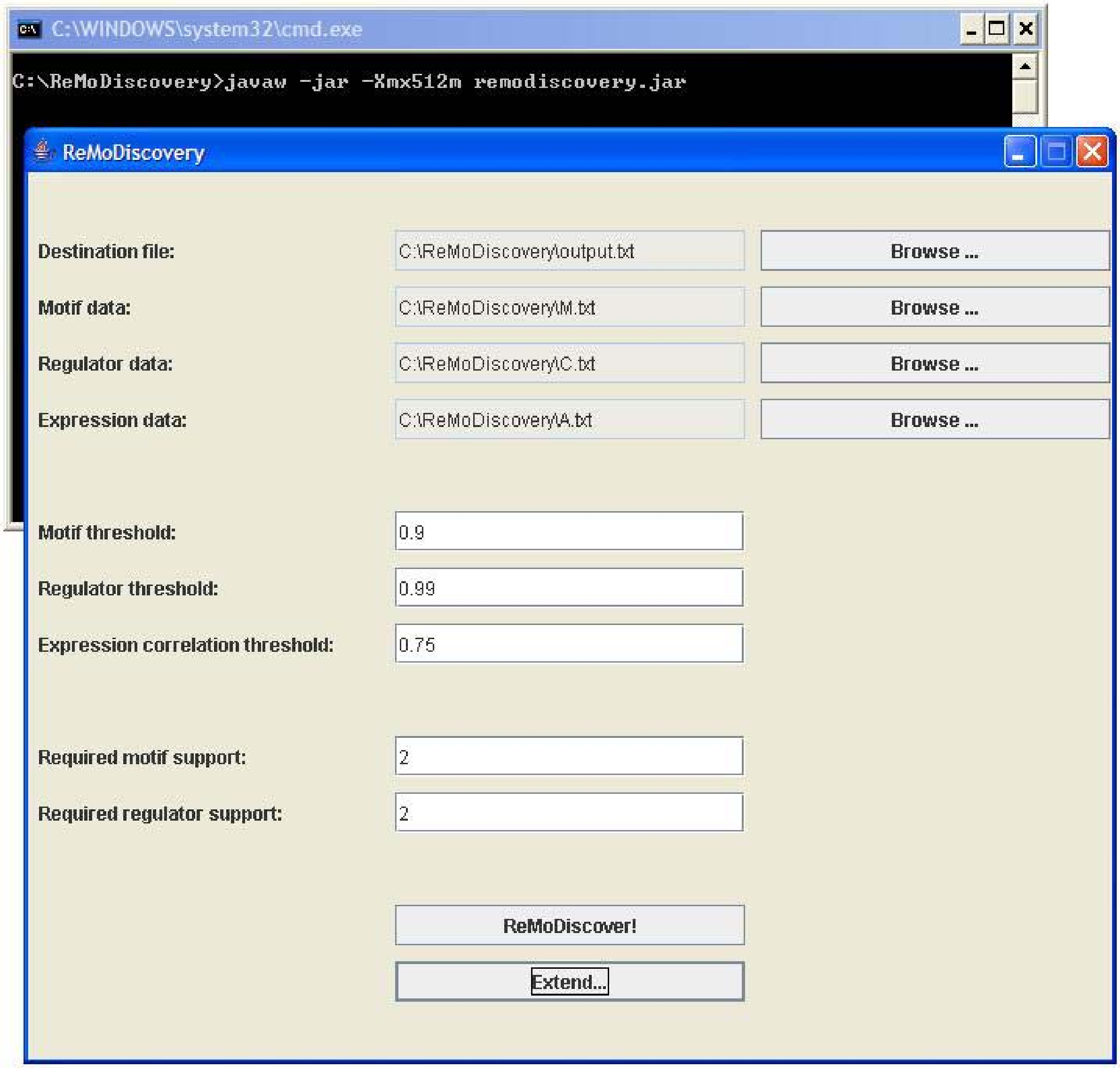

The following window appears:

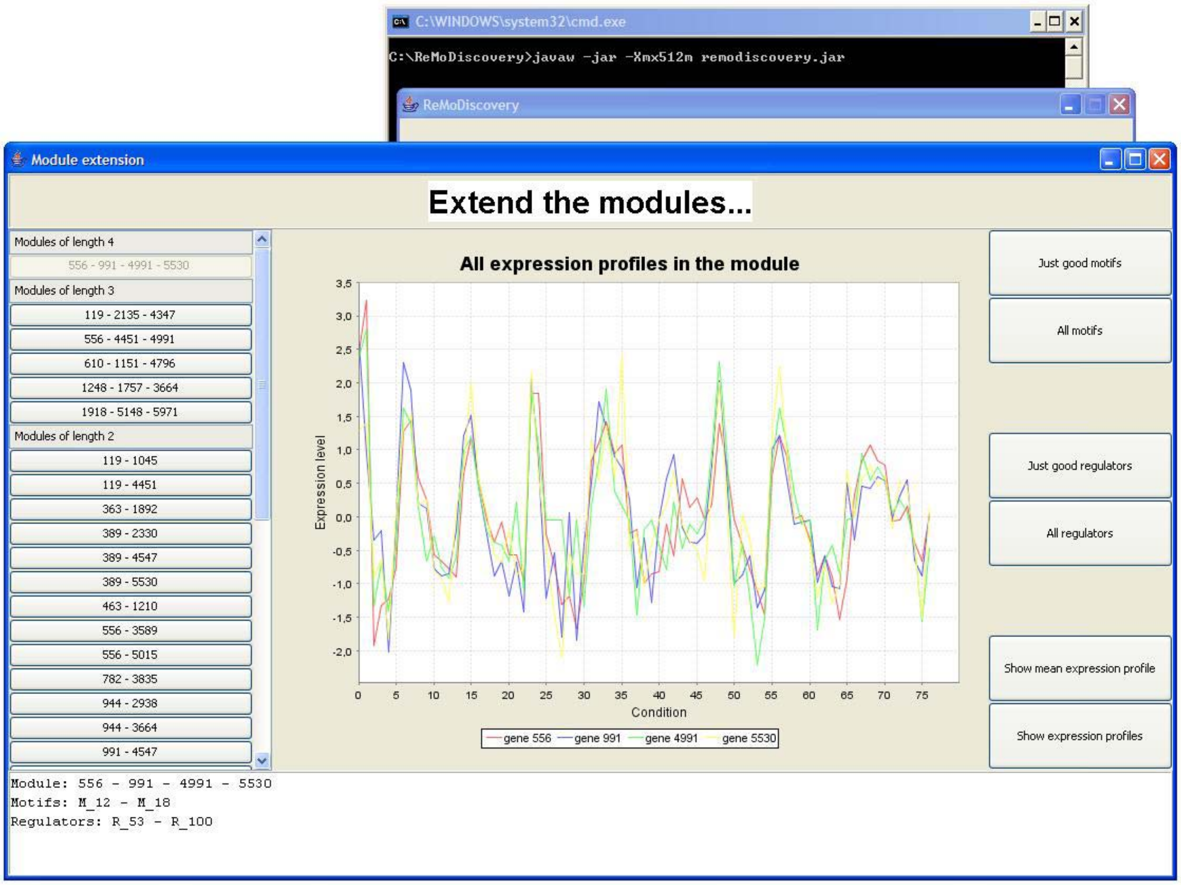

On the left side of the window the identified seed modules are shown. Modules are characterized by the genes contained within the module (indicated by their index), and by the motifs and regulators responsible for the corresponding regulatory program (indicated by their indices). The user can select the seed module of his interest. On the bottom of the window, the genes that are in the seed module and the regulators and the motifs of the seed regulatory program are shown. On the right side of the window you can ask to see the (mean) expression profile of the genes in the seed module, the *module enrichment p-value* plot for all regulators or for the regulators in the seed regulatory program and the *module enrichment p-value* plot for all motifs or for the motifs in the seed regulatory program.
